# Intelligent Fault Detection and Classification Based on Hybrid Deep Learning Methods for Hardware-in-the-Loop Test of Automotive Software Systems

**DOI:** 10.3390/s22114066

**Published:** 2022-05-27

**Authors:** Mohammad Abboush, Daniel Bamal, Christoph Knieke, Andreas Rausch

**Affiliations:** Institute for Software and Systems Engineering, Technische Universität Clausthal, 38678 Clausthal-Zellerfeld, Germany; daniel.bamal@tu-clausthal.de (D.B.); christoph.knieke@tu-clausthal.de (C.K.); andreas.rausch@tu-clausthal.de (A.R.)

**Keywords:** automotive software systems, fault detection and classification, deep learning, 1D-CNN, LSTM, real-time fault injection, hardware-in-the-loop (HIL), model-based testing phases

## Abstract

Hardware-in-the-Loop (HIL) has been recommended by ISO 26262 as an essential test bench for determining the safety and reliability characteristics of automotive software systems (ASSs). However, due to the complexity and the huge amount of data recorded by the HIL platform during the testing process, the conventional data analysis methods used for detecting and classifying faults based on the human expert are not realizable. Therefore, the development of effective means based on the historical data set is required to analyze the records of the testing process in an efficient manner. Even though data-driven fault diagnosis is superior to other approaches, selecting the appropriate technique from the wide range of Deep Learning (DL) techniques is challenging. Moreover, the training data containing the automotive faults are rare and considered highly confidential by the automotive industry. Using hybrid DL techniques, this study proposes a novel intelligent fault detection and classification (FDC) model to be utilized during the V-cycle development process, i.e., the system integration testing phase. To this end, an HIL-based real-time fault injection framework is used to generate faulty data without altering the original system model. In addition, a combination of the Convolutional Neural Network (CNN) and Long Short-Term Memory (LSTM) is employed to build the model structure. In this study, eight types of sensor faults are considered to cover the most common potential faults in the signals of ASSs. As a case study, a gasoline engine system model is used to demonstrate the capabilities and advantages of the proposed method and to verify the performance of the model. The results prove that the proposed method shows better detection and classification performance compared to other standalone DL methods. Specifically, the overall detection accuracies of the proposed structure in terms of precision, recall and F1-score are 98.86%, 98.90% and 98.88%, respectively. For classification, the experimental results also demonstrate the superiority under unseen test data with an average accuracy of 98.8%.

## 1. Introduction

Currently, software-driven automotive systems play an increasingly important role in daily life, notably in advanced functions such as Advanced Driver Assistance Systems (ADASs) [1]. As an interaction results between multiple subsystems and heterogeneous components, the advanced functions of an automotive software system (ASS) can be performed [2]. Along with the increasing complexity of software systems in automobiles, the probability of a failure occurrence also increases. A failure or defect in a software component of a safety-critical vehicle system, e.g., a braking system, can have a serious impact on human safety and infrastructure loss, leading to catastrophic incidents. Therefore, as a part of the quality assurance process, a rigorous reliability and safety assessment should be performed at each stage of the software development process [3]. According to the V-model [4], several test phases are conducted during the development process, including the executable model test, generated code test, implemented code in the real-time system test and real vehicle network test [5]. Each test phase is implemented and realized in a specific test platform, namely the Model-in-the-Loop (MIL) [6], Software-in-the-Loop (SIL) [7], Processor-in-the-Loop (PIL) [8], Hardware-in-the-Loop (HIL) [9] and Vehicle-in-the-Loop (VIL) [10].

A test drive is a well-known test approach performed in the real world to meet the functional safety requirements of ISO 26262 [11] such that unexpected faults can be detected. To this end, manufacturers conduct real-world test drives on public roads with prototype vehicles prior to production in which system behavior is recorded as multivariate time-series data [12]. There are significant limitations to this type of testing, however, such as a high cost per test kilometer, high time consumption, and high risk for the test driver [13]. Thanks to the vital role of the HIL platform in providing safe, flexible and reliable real-time validation of Electronic Control Units (ECUs), digital test drives can be carried out virtually in the laboratory, standardizing mileage and reducing costs tremendously [14]. However, the complex architecture of the ASS with its heterogeneous components, e.g., sensors, embedded computers, networks and actuators, generate a large amount of data. Thus, conventional approaches used by manufacturers to detect and classify faults in test drive recordings are time-consuming and depend on practical experience and expert’s knowledge, as mentioned in [15]. Consequently, an effective and intelligent method capable of detecting and classifying system-level faults in records generated by the HIL platform is required.

A deep neural networks-based approach has become an alternative to the traditional approaches, i.e., model-based and signal-based approaches, which neither need an accurate explicit analytic model nor provide a deep understanding and knowledge of the abnormal behavior of the signal symptoms. Nevertheless, it faces one complicating factor, i.e., collecting a representative training data set in terms of availability, cost and time consumption [16]. Specifically, when applying supervised Deep Learning (DL) methods, the availability of labeled data for model training presents a significant issue, where the system behavior under normal and potential abnormal conditions should be identified in advance and included in the recorded data. In the automotive domain, healthy data can be easily generated from test benches by recording the data in fault-free operations. However, collecting faulty data by recording under a faulty operation is a challenge [17]. Furthermore, in automotive systems, most of the potential hardware faults are random and occur as degradation of the component performance after years of operation.

Another complicating factor in the development of an automotive fault detection and diagnosis (FDD) model, i.e., fault identification, is that most proposed studies are relying on standalone DL techniques. Still, it has not been proven that a single DL method is sufficient for all FDD requirements. Consequently, there is an absence of a standard for selecting an appropriate technique from the wide range of the available DL algorithms to develop a robust and accurate model. In recent years, several studies have been conducted demonstrating that hybrid methods are superior to standalone methods, and much effort has been devoted to the study of FDD based on hybrid DL methods [18,19,20]. As a result, toward achieving a better performance of FDD in a complex system, combining different DL techniques as a hybrid method is comparatively an efficient solution. In this way, the advantages of each method can be utilized, and the limitations can be avoided. Thus, the research on developing an FDD model based on the hybrid DL methods is still in the initial stage, and further contributions in this field are needed.

To overcome the aforementioned drawbacks, in this study, we propose a novel FDD methodology, as a classification problem, for an HIL test of the ASS development. In particular, an intelligent faults identification model is developed and implemented based on hybrid DL techniques. The developed model aims at accurately detecting and identifying the fault types in automotive sensors and communication signals. To address the representative training data set problem, a novel real-time FI framework developed in the previous study [21] with an HIL simulation system was used covering most of the potential fault classes in the sensor signals of the ASS. By doing so, a faulty data set with complex test scenarios is simulated and recorded in real time, which is thereafter used for the training, validation and testing of the model. We demonstrate the capability and advantages of the proposed methodology using a complex automotive gasoline engine system as a case study, considering the vehicle dynamics with the environmental system model. Utilizing an unseen testing data set, the model performance was evaluated in terms of precision, recall, F-score and accuracy.

The main contributions of the study are summarized as the following:

- A novel methodology for developing a faults identification model of automotive sensor signals has been proposed to be used during the V-cycle of the ASS development process, i.e., the system integration testing phase. To this end, the integration of the LSTM and 1D-CNN is adopted to benefit from both classifiers and avoid their limitations.
- To overcome the lack of faulty data sets, the fault modes are simulated along with complex test scenarios without changing the original system model. For this purpose, the fault types have been programmatically injected in real time using a novel real-time FI with an HIL platform.
- In addition, eight different types of sensor and communication faults of vehicle signals have been considered as fault classes, i.e., spike, offset/bias, noise, hard-over, delay, gain, stuck-at and drift fault.
- To demonstrate the superiority of our proposed model, the classification performance with respect to each class has been compared with a standalone DL technique-based model, i.e., the LSTM and CNN technique.
- Finally, a high-fidelity entire vehicle model, i.e., an engine model, dynamic model, environment model and powertrain model, with realistic driving scenarios is considered to accurately and comprehensively represent the behavioral characteristics of the system.

The remainder of the paper is organized as follows. Section 2 introduces an overview of the background and related work, highlighting the main differences between the proposed methodology and previous research. The phases of the proposed methodology architecture are described in Section 3. In Section 4, the setup of the experiments is described, and the main steps of implementation are introduced. The results from evaluating the proposed model are discussed in Section 5. Finally, conclusions and future work are outlined in Section 6.

## 2. Background and Related Work

This section provides a brief overview of the background of the topics addressed. Specifically, the proposed methods and approaches for developing an FDD model are discussed. In addition, a review of the current literature on the FDC in automotive systems as an application domain is reviewed, focusing on the main findings and limitations of the proposed studies and highlighting the differences of our proposed methodology compared to the state-of-the-art.

### 2.1. Fault Detection and Classification Development Approaches

In the state-of-the-art, to develop an FDD model for multivariate time-series data, there have been several approaches proposed, i.e., model-based approaches [22], signal-based approaches [23], knowledge-based approaches [24] and data-driven approaches [25].

In dynamic systems, the model-based approach is considered the best option. Three different methods have been proposed for developing an FDD model, namely parity space-based [26], observer-based [27] and parameter estimation-based [28]. At the core is the idea of comparing the analytical redundancy of the developed mathematical model with the output data of the real physical system, and based on the residual signals generated, the faults can be detected and classified. In the automotive field, some studies have demonstrated the advantages and capability of the approach by proposing novel techniques for the FDD with high accuracy [29,30]. However, the expert knowledge required to build an accurate mathematical model and the increasing complexity of a modern ASS contribute to the difficulty of applying these types of approaches [31].

Although the signal-based approach is widely used in the real-time monitoring of industrial systems, especially in the steady state, it has some limitations. Due to the assumption that the collected signals contain significant information about the faults, expert knowledge of the symptoms of healthy systems is required to detect the presence of faults when analyzing the measured signals [32]. The extracted features of the signals in this approach are categorized into three domains, namely the time domain [33], frequency domain [34] and time–frequency domain [35].

A knowledge-based approach does not require pre-classified training sets [36]. However, due to the lack of specific qualitative expert knowledge and the complexity of modern vehicles, the knowledge-based approach is limited and insufficient to build an FDD system [37]. Besides the extensive human intervention required, nonlinear relationships between signals in the complex system render the detection and classification of the faults difficult.

Above all, the introduction of advanced sensor technology, the rapid development of computational methods, the availability of sufficient computational resources and raw information from measured signals, and the simplicity of model realization with its high efficiency played a crucial role in drawing researchers’ attention to data-driven approaches as a means of FDD [17]. The main idea of this approach is to use the constructed knowledge from high-quality historical data to detect and classify the abnormal behavior. In accordance with [24], FDD methods can be classified into statistical analysis data-driven and non-statistical analysis data-driven. The most popular methods of the statistical approach are PCA [38], ICA [39] and PLS [40]. The pattern recognition-based Machine Learning (ML) techniques [41], on the other hand, are often used in the non-statistical approach because of their adaptive learning and nonlinear approximation capabilities. Depending on the tasks to be performed, i.e., clustering, classification, regression and anomaly detection, ML techniques are classified into unsupervised learning methods [42], semi-supervised learning methods [43], supervised learning methods [44] and reinforcement learning methods [45].

DL [46], as a subarea of ML, has been gaining importance in developing complex FDD models based on neural networks that employ a large number of layers, especially when multiple data streams are involved [47]. The reason behind that is its ability to outperform traditional statistical ML methods and overcome their limitations in terms of feature extraction, computational cost and dimensionality reduction. Not only that, the rapid development of Graphics Processing Units (GPU) paved the way for the more efficient processing of huge amounts of data, which in turn contributes to achieving the outstanding performance of DL-based approaches. Therefore, in the last decade, the interest in the development of FDD systems using DL techniques has increased, and various DL architectures, e.g., Deep Belief Network (DBN), Restricted Boltzmann Machine (RBM), Convolutional Neural Network (CNN), Recurrent Neural Networks (RNNs), Autoencoders (AE), have been proposed for different application domains. A comprehensive survey of the DL architecture focusing on current challenges and future developments can be found in [48].

In the automotive domain, several studies have been proposed for the utilization of DL for various tasks, e.g., for anomaly detection, fault classification and fault prognosis. As an application area, some focus on safety-critical vehicle systems, i.e., engine, powertrain, steering and suspension, while others focus on fuel cells and batteries of electric vehicles. For example, in [49], You et al. proposed a data-driven framework based on the LSTM aiming at diagnosing the state of electric vehicles. While using driving data, a CNN-based FDC of suspension systems, i.e., automotive damper defect diagnosis, was proposed in [50]. Moreover, to develop an FDD model for a fuel cell electric vehicle (FCEV) powertrain, an artificial neural network (ANN) is used in [51] to isolate the faults, i.e., drying and flooding faults in a single cell, as a classification problem based on the variation of temperature and current density. In [52], an FDC method for vehicle drive systems based on DL, i.e., hybrid DBN, was proposed, where the new data fusion method was investigated focusing on automotive gearbox faults. Toward classifying the faults in the braking system of a vehicle and performing the condition monitoring, a clonal selection classification algorithm was proposed in [53] as an artificial intelligence (AI) technique. The model is trained based on the vibration signals collected under healthy and faulty conditions.

### 2.2. Convolutional Neural Network (CNN)

Nowadays, among numerous ML algorithms such as support vector machine (SVM) and decision tree, DL algorithms, especially CNNs, have become the preferred networks for FDI. Compared to other traditional manual methods, a useful property of CNNs is their ability to automatically extract features, a key step in the learning algorithm, resulting in a reduction in time and effort in the training process. Beyond the successful achievements of CNNs in specific applications with 2D data, such as images and speech recognition, a 1D time-series-based CNN has also proven to be successful in the FDD area with high performance in terms of accuracy and training time. In a complete sentence, surveying the CNN algorithms and their applications for FDD, i.e., fault size detection, identification and estimation, have been investigated in [48,54]. Other proposed studies to employ the 1D-CNN for the FDC of rotary machines can be found in [55,56,57].

A CNN is a DL model based on the ANN architecture in which additional layers are added to overcome the drawbacks of the ANN. Feature extraction and classification are the two main phases of a CNN [58]. With the goal of automatically extracting and learning features from raw data, the feature extraction part includes an input layer, convolutional layers and pooling layers stacked in the form of a network. In the classification part, the features from the last pooling layer are received at the fully connected layers, and based on the learned features from the previous layers, the classification tasks are performed.

Mathematically, convolution and pooling are expressed in Equations (1) and (2), respectively.

(1) $$f{\left(y\right)}_{i}=AF\left(\Sigma \;w\left(i,j\right)\times y\left(i\right)+b\left(i\right)\right)$$

(2) $$x_{i}=Pool_{\left(m,n\right)}\left(f{\left(y\right)}_{i}\right)$$

where AF is an activation function like Sigmoid and ReLU, y(i) is the *i*th signal features, w(i,j) and b(i) represent weight and bias, $f{\left(y\right)}_{i}$ is the output feature extraction and (m,n) represents the *n*th pooling area with size *m*.

### 2.3. Long Short-Term Memory (LSTM)

One of the first studies presenting the deployment of the LSTM networks is reported in [59]. The key feature of the LSTM architecture is its ability to learn data with long-term dependencies. LSTM, as an extension of a Recurrent Neural Network (RNN) [60] with the ability to remember past information, overcomes the disadvantage of the RNN reflected in gradient vanishing and gradient explosion. Consequently, LSTM has been used for a wide range of complicated sequential problems due to its high performance compared to other deep neural networks in terms of feature learning and classification results, notably in the case of the dynamic information of time sequences. For instance, with respect to fault monitoring, LSTM has been used to develop an FDD model for the Tennessee Eastman (TE) process in [61]. Similarly, in the Unmanned Aerial Vehicle (UAV) field, the detection of sensor anomalies using LSTM was investigated in [62]. Regarding the diagnosis of bearing faults in electrical machines, an improved method based on the integration of the LSTM and CNN techniques into a unified structure was proposed in [63], achieving an average accuracy of 99% over the test data set. Last but not least, as far as automotives are concerned, several studies have been proposed to explore new methods for FDD using either standalone LSTM or through the integration of the LSTM with other DL techniques, targeting different automotive systems, e.g., faults in engines [64], EV batteries [65] and fuel cell vehicles [66].

**Figure 1:**
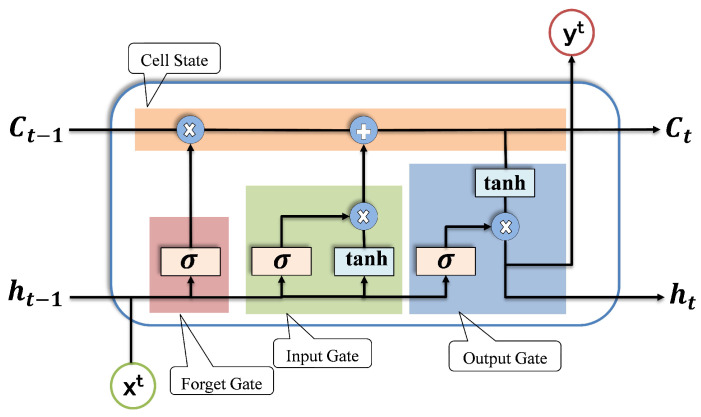
Internal structure of LSTM cell.

The key element of the LSTM is the chain units called the cell network. Three different gates, namely forget, input and output, constitute the internal structure of the cell and are connected to the cell state in a specific fashion, as illustrated in Figure 1. The state of a cell is represented by the horizontal line at the top of the cell. It is likened to an assembly line as it runs along the entire chain of the cell. The LSTM is able to update the cell state using gates. Deciding what information to eliminate from the cell state is determined by the forget gate. On the other hand, the input gate is responsible for deciding what new information should be stored in the cell state. For this purpose, the Sigmoid is computed to decide which values will be updated and then Tanh creates a vector of new candidates that can be added to the cell state. Subsequently, Tanh and Sigmoid are combined and added to the cell state.

Mathematically, Sigma and Tanh are represented in Equations (3) and (4), respectively. Equation (5) applies to the cell state update, where $g_{t}$ and $d_{t}$ represent Sigma and Tanh output, respectively. $C_{t}$ is the output of current state and $C_{t-1}$ represents the output of previous state.

(3) $$g_{t}=\sigma \left(W_{g}.\left[h_{t-1},x_{t}\right]+b_{g}\right)$$

(4) $$d_{t}=tanh\left(W_{d}\left[h_{t-1},x_{t}\right]+b_{d}\right)$$

(5) $$C_{t}=\left(f_{t}\times C_{t-1}\right)+\left(g_{t}\times d_{t}\right)$$

The value of the gate depends on $h_{t-1}$ and $x_{t}$. $h_{t-1}$ is the output of the previous state, where $f_{t}\in 0,1$. $f_{t}$ is further multiplied by the current state value. If $f_{t}=1$, the state of the cell is fully maintained, otherwise it partially depends on the value.

Finally, the output of the cell is determined by the output gate. With Tanh of the cell state, the output is calculated and then multiplied by the output of the Sigmoid. Mathematically, Sigma in the output cell is shown in Equation (6), and the output of this gate is shown in Equation (7)

(6) $$O_{t}=\sigma \left(W_{O}.\left[h_{t-1},x_{t}\right]+b_{O}\right)$$

(7) $$h_{t}=O_{t}\times tanh\left(C_{t}\right)$$

### 2.4. Fault Types

**Figure 2:**
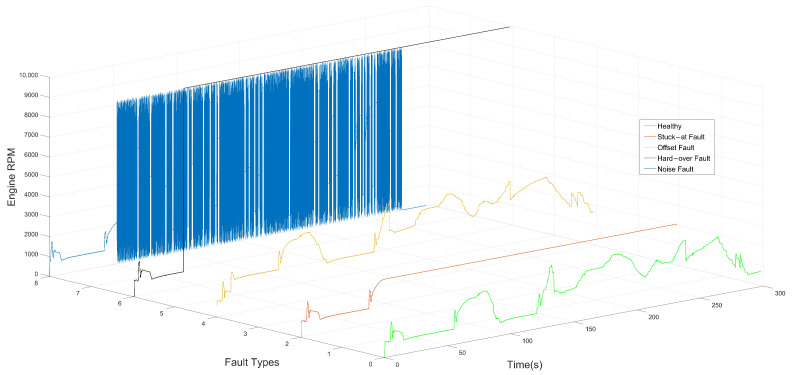
Healthy signal and fault types: Stuck-at fault, Offset fault, Hard-over fault, Noise fault.

**Figure 3:**
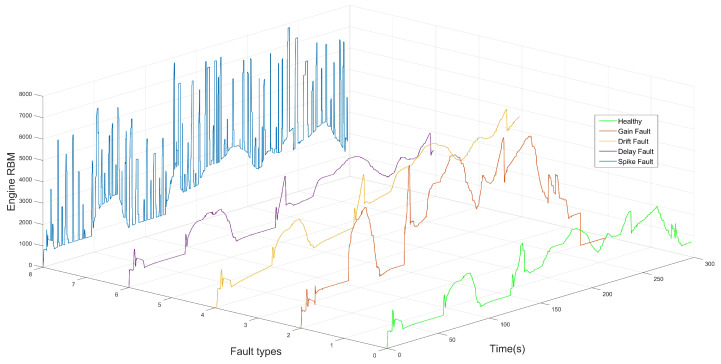
Healthy signal and fault types: Gain fault, Drift fault, Delay fault, Spike fault.

The major factor behind the selection of the appropriate classes of fault classification or identification models, based on DL, is the specification of the fault types. Based on recent studies in the literature, faults can be classified into four categories, namely hardware faults [67], software faults [68] and network and communication faults [69]. Each category, in turn, can be divided into subclasses. On the basis of the type of components that contain the defect, i.e., sensors, actuators, a plant or control system, faults are grouped into hardware faults. In contrast, bit fips, runtime errors, processor register corruption and target restart are types of software faults. Communication-related faults involving delays, data loss and out-of-order delivery belong to abnormal states of communication protocols that occur on the network path between components, applications or subsystems. There are also other categories of faults, which can be classified as transient, permanent and incipient, depending on the duration and type of occurrence [70]. In signal-based system behavior, i.e., time-series data, several types of faults have been identified in [71,72,73,74], which take the form of measurement offsets, stuck-at or scaling from true values. Gain, delay, stuck-at, hard-over, spike, noise and drift faults are some examples of sensor and communication faults, as can be illustrated in Figure 2 and Figure 3, respectively.

In this study, due to the significant impact of random hardware faults on the safety of the ASS, sensors and communication faults have been considered in terms of signal behavior. The mathematical representation of the aforementioned fault types can be summarized in Equation (8) and Table 1:

(8) $$y\left(t\right)=d_{v}x\left(t\right)+o_{v}$$

where y(t) is a faulty or manipulated signal value, $d_{v}$ represents the gain value and x(t) is the healthy or standard signal value. $o_{v}$ represents the offset/bias value.

It is also worth noting that a variety of factors can cause the above-mentioned sensors and communication faults. Based on [75,76], dirty or deteriorated sensors, aging, corrosion, vibration, electromagnetic interference, improper calibration and weak batteries are good examples of direct causes of automotive sensor faults.

### 2.5. Related Work

Tackling the problem of the high cost of collecting representative data sets in the automotive industry, several studies have been proposed to develop an FDD model relying on either real-world or simulation platforms to collect measurement data in different modes of system operation, i.e., faulty and non-faulty modes. Safavi et al. [77], for example, have proposed a DL algorithm-based fault detection, isolation, identification and prediction system targeting sensor faults in autonomous vehicle systems. In the proposed study, the real data of three sensors, i.e., the accelerator pedal (AccP), steering wheel angle (SWA) and brake pressure (BP) sensor, collected from an automobile bus and recorded on highways, have been used for training, testing and validating the proposed fault detection and isolation (FDI) system. Specifically, the collected autonomous driving data set includes approximately 35 min of real driving scenarios, i.e., in the city and on the highway, at three different recording locations, namely Ingolstadt, Munich and Gaimersheim. Besides the healthy real data set, a faulty data set is generated using the FI method by capturing the system behavior in the faulty mode. The results of the study showed the capability of the proposed fault detection system with an accuracy of 99.84%, while the sensor fault identification system had an accuracy between 73.00% and 100.00%. However, the sensor faults injected into the healthy data set are limited to four fault types, namely drift, hard-over, erratic and spike faults, whereas eight different fault types have been considered in our proposed methods, increasing the coverage of fault occurrence, not only in the sensors but also in the communication signals. Another difference resides in the method of the FI used to collect the faulty data. In the study, a normal data distribution with a standard deviation was statically used, while in our research, a real-time FI framework was used to generate faulty data, and hence study the effect of faults on the system’s behavior, considering the real-time constraints and the simulation of the whole-vehicle system with high fidelity. Similarly, in [78], Theissler proposes an anomaly detection approach using an ensemble classifier to analyze vehicle test records based on data recorded during on-road test drives. Albeit both known and unknown faults can be effectively detected by the proposed system with good robustness to different fault types and driving scenarios, the proposed system is limited to a detection problem with only two classes, i.e., the binary classification of inputs into healthy or faulty. In contrast, in our proposed method, besides the detection task, the identification of the type of detected fault is also explored as a classification problem with nine different classes (healthy and faulty). On top of that, using real data from a vehicle prototype as the basis for the training data set is costly and poses a high risk to the driver, especially when injecting faults while driving to validate the proposed system, whereas in our proposed method, this problem has been addressed by using HIL simulations with real-time FI.

To overcome the limitations of using a real vehicle prototype, some research that has been conducted relies on the real automotive system test benches as the solution for the training data set acquisition. For example, in [79], a probabilistic fault classification method of time-series data was proposed, aiming at classifying unknown faults. To this end, a combination of Weibull-calibrated OSVM and Bayesian filtering have been employed for modeling fault classes and fault classification, respectively. To demonstrate the proposed method, an internal combustion engine was used as a case study. Meanwhile, to generate the residual data set, a real engine test bench was used with its model covering seven different types of engine faults, i.e., sensor faults, leakage and air filter clogging. However, to operate in a realistic environment, a highly accurate mathematical model is required, which drives up the cost in terms of complexity, whereas our proposed method uses a real-time simulation platform (HIL System) to inject the faults and capture the system-level effects. In the same context, but for a different application, Kaplan et al. [80] used an electric vehicle prototype to test their proposed LSTM-based FDD approach. However, to collect the faulty data, different fault scenarios, i.e., short-circuit and open-circuit faults, are injected into the simulated EV system in Matlab/Simulink without considering the real-time conditions, but in our study, this problem was addressed by injecting the fault in real-time with an HIL. Aiming at tackling both single and simultaneous fault diagnosis issues of automotive engines, a novel framework based on the combination of three phases, i.e., feature extraction, probabilistic reject machine (PCM) and decision threshold optimization, has been proposed by Zhong et al. [81]. As an experimental platform, a real sports car with an ECU, onboard oxygen sensor, ignition pickup and microphone have been used to collect the data set under various faulty conditions, focusing on three engine signals, namely the air ratio, ignition pattern and engine noise signal. However, to avoid the high cost and risks of collecting real data sets from a real vehicle, the HIL simulation functions with Rapid Control Prototyping (RCP) have been used in our study.

One more possibility to obtain a representative data set of healthy and faulty operation modes is to employ a simulation platform. As an example, in [82], researchers propose that the MATLAB/IPG CarMaker co-simulation platform can be used to model sensor faults and collect the resultant data set; in this way, an architecture for fault detection, isolation and identification can be developed for multiple faults in autonomous vehicles. Notwithstanding that the proposed FDI architecture using SVM is of high accuracy, 94.94% and 97.01%, respectively, the faults have been injected at the model level without considering real-time constraints.

In the last decade, the advancement of the HIL simulation with its features has attracted the attention of both academic and industrial researchers. Rather than real vehicle hardware elements, the HIL platform’s ability to fidelity simulate a complex nonlinear system enables effective and accurate analysis, verification and validation of an ASS in real time. Therefore, using HIL systems, considerable attention has been paid not only to the development of control software modules but also to the verification and validation of the developed systems [83]. Beyond the advances in a real-time simulation with HIL, the development of the intelligent FDC of complex software systems has attracted much attention. As reported in the literature, several methods have been proposed with the aim of approaching this problem. Employing the HIL system, an integral sensor fault diagnosis method for railway traction drives has been proposed, for example, in [84]. This was accomplished by using a combination of model-based and data-driven techniques to develop a model capable of detecting, isolating and evaluating the sensor faults, i.e., gain and offset faults. However, to simulate the system behavior in the presence of faults, the system model has been extended by adding additional model blocks, which, in turn, affects the real-time properties of the control task. In contrast, in our proposed work, all the studied faults in automotive sensor signals have been programmatically injected without modifying the original system model. Moreover, the classification model in the mentioned study is limited to three classes and depends on the manual’s threshold setting, while nine different classes were considered in our proposed method relying on DL-based classification. Concerning the same platform for identifying the faults in the air brake system of heavy-duty vehicles, a multiclass classification model based on the wheel speed sensor data collected in an HIL simulation system was proposed in [85]. Compared with the random forest-based model developed in the study, the model we proposed based on hybrid DL techniques exhibits better classification than the traditional ML method. In addition, to collect the faulty data during the test scenario, a real driving system with four wheels connected to an HIL simulator was used to simulate the system behavior under faulty conditions, whereas the method proposed in our work depends on the entire vehicle system model deployed into the HIL simulator and connected to the control system. Finally, Namburu et al. [86] have proposed an FDD method based on data-driven pattern recognition techniques, i.e., support vector machines. The focus of the proposed method is on automotive engine faults, i.e., eight engine faults with different severity levels inserted into the engine system model, which was simulated in the real-time ComputeR Aided Multi-Analysis System (CRAMAS) simulator. Despite the fact that the model of the proposed method accomplishes high diagnostic accuracy in terms of detecting, isolating and estimating the severity of faults covering high potential fault locations, there are significant differences to our proposed method. The faults in the study were injected as incremental changes at different locations to simulate the engine functionality under different scenarios and different fault conditions, whereas in our proposed study, the focus was on the fault types rather than the fault locations. Furthermore, the data collected in the study are limited to test the feasibility of the presented approach without considering the fault types and the realistic driving scenarios, whereas in our proposed study, eight different fault types were considered and a whole-vehicle model with high-fidelity simulation was used. An overview of the related work is given in Table 2.

It can be summarized that the traditional way to obtain data containing faults is obtaining the faulty data set from the automotive industry. However, manufacturers will probably not disclose their highly confidential defective data. On the other hand, real test drives enable high test coverage, but test costs increase as test mileage increases. Consequently, such a recorded data set is still limited and expensive. Moreover, as the relevant scenario and critical driving situation are defined, the probability of risk to the test driver also increases. Therefore, it is a challenge to have access to confidential industrial data and to obtain data that contain faults from real test drives, and this problem has not yet been sufficiently explored. The novelty of this study is to develop an FDC methodology for the HIL real-time testing of an ASS during manufacturing before the operation mode, i.e., in the system integration test phase, considering the entire vehicle system models. To this end, a representative faulty data set is generated by programmatically injecting nine different fault types into the HIL system in real time without modifying the original system model. Through this approach, a novel intelligent FDC model is developed based on hybrid DL techniques, i.e., the LSTM and CNN. The characteristics of the selected techniques, e.g., their ability to process large amounts of data, the automatic feature extraction from the multivariate time-series data and their ability to build an accurate FDC model with prior knowledge, were the reasons behind selecting such methods. Furthermore, by leveraging data from a real-time FI in the HIL, and considering real-time constraints and a whole-vehicle system model, the cost of the data acquisition is reduced, a high-fidelity simulation is ensured and a model for the test-drive data analysis with high accuracy is developed.

## 3. Methodology

### 3.1. Hybrid DL-Based Fault Detection and Classification Methodology

**Figure 4:**
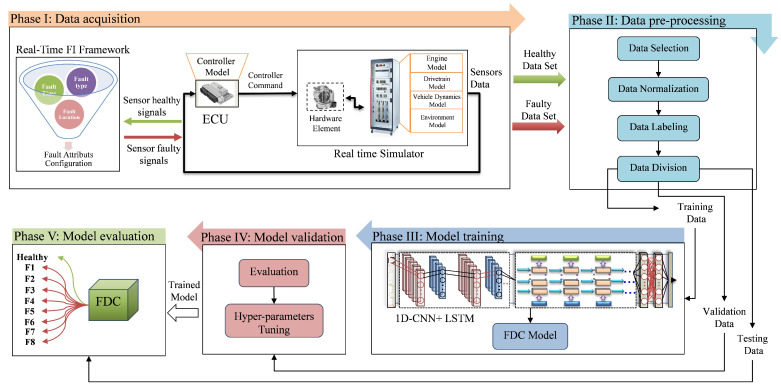
Hybrid DL-based FDC methodology.

The framework of the proposed FDC method includes five different phases, namely data acquisition, data pre-processing, model training, model validation and model evaluation, which together form the steps to develop the target model, as shown in Figure 4.

#### 3.1.1. Phase I: HIL-Based Data Set Acquisition

According to the supervised learning approach, a labeled training data set is required as a prerequisite for developing an FDC model that can classify the system state into known fault types and categories. Traditionally, to simulate the system behavior under fault conditions, the system model of the controlled plant and controller are extended to include additional blocks, which in turn affects the real-time constraints. To overcome this drawback, the proposed methodology uses real-time FI method with the objective of generating a representative data set. By doing so, most of the fault types in sensor and communication signals can be covered without modifying the original system model. The main components of the data acquisition phase are the real-time FI framework and the HIL system.

#### 3.1.2. Real-Time Fault Injection Framework

To generate a representative faulty data set under different conditions, the real-time FI framework developed in the previous study [21] is employed. The framework is capable of programmatically injecting eight different types of faults into a complex system, such as a vehicle system with high-fidelity simulation, in real-time without altering the system model. By doing so, real-time constraints are taken into account and the majority of common faults in automotive software signals are covered. FI framework used in this methodology includes four key components, i.e., HIL user environment, HIL system real-time configuration, FI framework and HIL system. Meanwhile, in order to effectively collect faulty data using the FI framework, three main attributes should be configured by the user, namely fault type, fault location and fault time. According to Theissler [78], the potential fault locations in an in-vehicle network are functional specifications, network, sensors, actuators, ECUs, gateways, a power supply, vehicle subsystems and a data acquisition system. In this study, sensor and communication signals are considered as the target locations of FI. In terms of fault types, a list of potential hardware fault types that could occur in the vehicle’s software system are provided in the fault library. There are eight different types of sensor and communication faults considered in this study with respect to signal behavior, i.e., delay, gain, offset/bias, noise, hard-over, spike, stuck-at and drift faults. Finally, the fault time point is the time at which the fault is injected. The fault instant can be defined based on the driving cycle so that the tester can inject the fault according to the desired time. Once the fault attributes are defined, the fault injector manipulates the healthy signal from the CAN bus accordingly in real-time.

#### 3.1.3. HIL System

Compared to the conventional simulation tools, HIL simulation provides an efficient, fast and realistic simulation in real time with high accuracy [83]. Two main components make up the structure of the HIL system, namely the HIL real-time simulator and the real ECU. In the HIL simulator, the system models, including engine, powertrain, environment and dynamic vehicle models, are deployed and executed. Meanwhile, the control logic, which is represented as a controller model, is implemented and executed in the real ECU. The connection between them is realized using the CAN bus as the communication protocol. Thanks to the CAN bus, the control commands from the ECU to the actuators as well as the sensor signals from the plant to the ECU can be transmitted so that the action is executed according to the control logic. Additionally, other real elements of the vehicle system, e.g., sensors, actuators, steering wheel and pedals, can be connected to the HIL simulators via electrical interfaces. After fault injection, the data are recorded at the system level using HIL tools in different file formats depending on the experimental setup. Subsequently, these recorded files are manually converted to CSV format, where the pre-processing phase takes place.

#### 3.1.4. Phase II: Data Pre-Processing

Data pre-processing is an essential step toward the development of DL models because irrelevant features in the training data can have a negative impact on the classification accuracy [87]. Moreover, a large amount of useless features leads to increase the cost of implementing DL algorithms in view of the high computational overhead. Therefore, pre-processing data sets in advance contributes to avoiding an overfitting problem and reducing computational costs. Another major challenge in developing an effective intelligent FDD is the imbalanced data set, especially in real-world applications. The ratio between healthy and faulty data is not typically equal, which in turn negatively affects the model performance. For this reason, several approaches have been proposed to address this problem, namely augmentation-based, feature learning-based and classifier design-based approaches [88]. In this study, permanent faults have been injected such that the percentage of faulty data classes is sufficient compared to the healthy data. Meanwhile, to solve the problem of unbalanced data distribution among the faulty classes, the feature learning-based CNN with a large data set was used to effectively learn the features of unbalanced faulty classes. In this way, the feature extraction and learning capability of CNN can be employed to reduce the risk of overfitting when the distribution of the faulty data set is different. Notably, in the case of transient fault injection, a modification of neural networks is required to handle the small and unbalanced faulty data set, e.g., using a regularized CNN.

In the proposed methodology, the pre-processing phase is divided into four steps, namely data selection, data normalization, data labeling and data division. The first step after data collection is to select the relevant features so that useful system variables can be efficiently included in the training data, based on which the classification task is performed. In this study, five different variables at system level were selected to be used in the classification task. The selected variables are engine RPM, engine torque, vehicle speed, rail pressure and intake manifold pressure. The next step after selecting the system variables is data cleaning and normalization. Noteworthy, due to the uncertain data collected from the real system, cleaning and filtering of the data with respect to outliers and missing values are required before feeding it into the learning algorithms. However, this step is not necessary for the simulation data collected from the HIL platform, as in the case of this study. On the other hand, with the purpose of improving the performance in training network, the selected data are converted into a binary matrix. To this end, the Z-score normalization function [89] is used to rescale the amplitude of the variables such that the data values are uniformly in the range [0–1]. Once the data are normalized, the time-series samples are labeled with the corresponding classes to support the supervised learning task. This is accomplished by labeling each category’s data set with a label that refers to the healthy class or a specific fault class. In this study, nine different classes are labeled to cover most fault types, including non-faulty data. Finally, the data set is divided into three subsets to be used later in different phases, i.e., training, validation and testing. In particular, 60% of the whole data set is dedicated to the training process. While the rest of the dataset is used for the purpose of validation and testing, 20% is allocated to each phase, respectively. To overcome the drawbacks of traditional feature extraction, which depends on domain knowledge and human experience, in the proposed methodology, features are automatically extracted from training samples based on powerful data processing ability of deep neural networks. Specifically, by employing feature extraction capability of CNN [90], deep features of faulty and healthy input data are effectively captured through the learning process within the hidden layers.

#### 3.1.5. Phase III: Model Training

**Figure 5:**
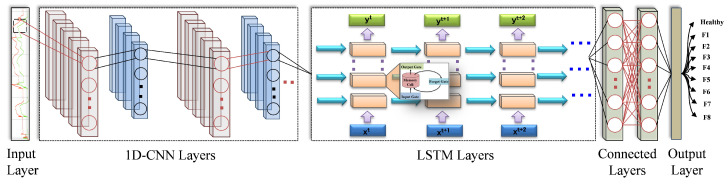
Deep neural network architecture of the proposed model.

The key component of the proposed methodology is to design the architecture of the deep neural network. In addition, the identification of the hyperparameters of the selected network architecture plays a vital role in the development of a highly accurate model. The literature on FDD based on DL shows a variety of approaches corresponding to different domains. Among the proposed DL models, CNN and LSTM achieved fruitful remarkable achievements to build intelligent diagnosis models. Because each type of fault has an individual effect on the system data, several important features are derived in the data at different times. As a result, thanks to the ability of CNN to reveal significant features of data without human supervision, the CNN algorithm is an optimal option for developing FDC models. However, its drawback in processing multivariate time-series data motivated to investigate other algorithms. On the other hand, LSTM and Gated Recurrent Unit (GRU) have been introduced to overcome the limitations of RNN, i.e., vanishing and exploding gradients. Moreover, in the context of sequential time-series data, LSTM and GRU surpass other techniques from the viewpoint of accuracy. Despite the advantages of GRU reflected in its low required resources and fast inference time compared to LSTM [91], LSTM has achieved better accuracy on larger data sets, as indicated in [92]. In addition, LSTM have exhibited remarkable performance in classification tasks in [93,94,95]. Therefore, the structure of recurrent units can be effectively selected depending on the data set and corresponding application [96]. In this study, to achieve accurate fault classification, LSTM is employed to cope with dynamic multi-mode behavior with large data sets. However, for complex multiclass classification tasks, high overhead is required in terms of cell structure and training time. Aiming at overcoming the limitations of both techniques, i.e., CNN and LSTM, and benefiting from both classifiers, we have employed the integration of both structures to develop the target model, as can be seen in Figure 5.

The input layer, 1D-CNN layers, LSTM layers, the fully connected layer and the output layer are the main components of the proposed network architecture. The training data set in the form of time-series data is fed into the 1D-CNN network through the input layer. In particular, after the row data are received by the input layer, local features are extracted by the convolutional layer and stored as feature maps. Feature maps contain different nodes associated with a particular input region. Similarly, several nonlinear activation functions exist to extract features from the input layer. Among them, the most commonly used functions are the Rectified Linear Unit (ReLU), hyperbolic tangent and Sigmoid function. According to the results of the conducted research on the development of intelligent FDD of machines, it has been concluded that the highest test accuracy was achieved with the ReLU activation function compared to Sigmoid and Tanh [97]. Moreover, during the training process, a significant improvement in DNN behavior can be observed when using ReLU compared to Sigmoid [98]. Therefore, after trying the aforementioned activation functions, ReLU was selected for the proposed architecture to obtain a nonlinear expression and provide better discrimination of the learned features.

The key role of the pooling layer is reflected in the reduction in the feature map, which is located after the convolutional layer. Max pooling, average pooling and L2 norm pooling are the most common methods used in this layer. Rather than connecting the last pooling layer of the CNN to the fully connected layer, in this study, a multi-layer LSTM network is designed to be after the CNN layers. By doing so, the features are further processed, paving the way for the fully connected layer to calculate the probability score for each class. Subsequently, the features of the last LSTM layer are processed by the fully connected layer so that the probability score of the output is generated for classification. Finally, at the output layer, the class with the highest probability is considered the output, which is either related to the type of the detected fault or to non-faulty data. Table 3 depicts the specification parameters of the proposed network architecture.

#### 3.1.6. Phase IV: Model Validation

Using the validation data pre-processed in Phase II, the trained model from Phase III is validated in terms of accuracy. Based on the validation results, the specifications of the hyperparameters of the network structure are adjusted so that the FDC model is optimized. To be specific, the value of the loss function is reduced, and the accuracy is increased. During the validation phase, the main hyperparameters considered are learning rate, the number of epochs, batch size, the number of batch normalization, CNN, LSTM, dense and drop layers.

#### 3.1.7. Phase V: Model Testing

Once the developed model is optimized, i.e., the hyperparameters are tuned based on the validation results, the final model with the optimal hyperparameters is tested in this phase. Employing the unseen data prepared in Phase II, the performance of the optimized FDC model is evaluated. To this end, various evaluation metrics can be used, such as precision, recall, F1-score and accuracy [99]. The objective of the model is to detect and identify faults, as a classification problem, in recordings of the HIL test. More specifically, eight different types of sensor and communication faults along with healthy data are to be correctly detected and identified. In this study, the performance of a hybrid 1D-CNN and LSTM is presented in comparison to a single CNN and LSTM, respectively.

## 4. Case Study and Experimental Implementation

### 4.1. HIL System

**Figure 6:**
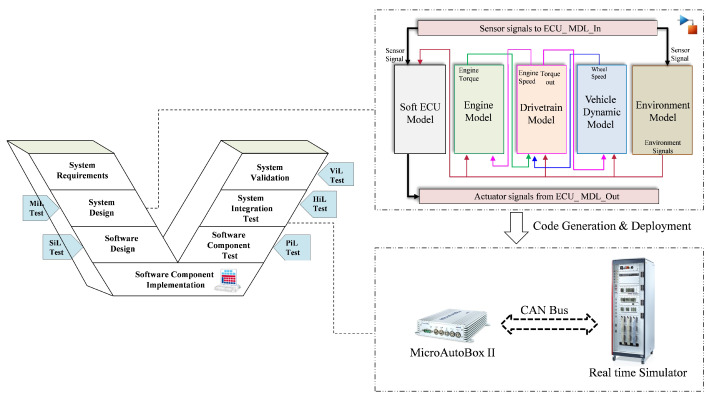
System architecture of the case study with different test phases of V-model.

**Figure 7:**
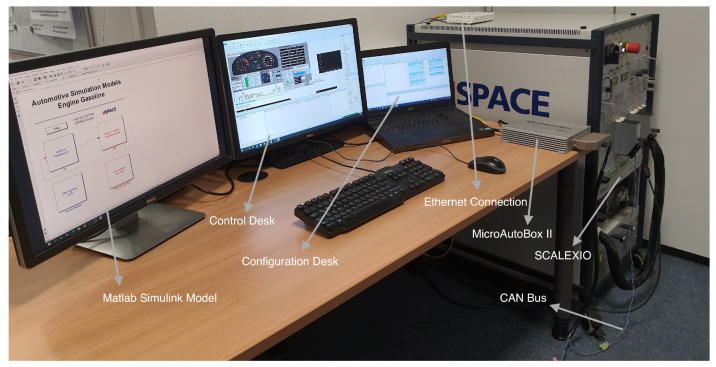
Scheme of the complete HIL simulation system.

As a case study, the ASM gasoline engine system, combined with the dynamic vehicle model from dSPACE [100], is used to demonstrate the applicability of the proposed methodology and to verify the performance of the proposed model. Additionally, by using the HIL system, healthy and faulty data sets of training, validation and testing are generated in real-time during the operation. Figure 6 illustrates the architecture of the experimental platform used in this work. As can be seen, to achieve high simulation fidelity and to capture the comprehensive characteristics of the system behavior, environmental system models of the case study are included. Specifically, the powertrain, vehicle dynamics and environmental system are modeled and simulated along with the engine system. In addition, in case no real ECU is available, the softECU is employed to enable the simulation of the control logic. Fundamentally, the control system is modeled separately and implemented in the real ECU. To realize the connection between the real ECU and the HIL simulator, I/O blocks are employed to simulate and manage the signals from and to the ECU. The aforementioned systems are modeled and simulated in the MATLAB/Simulink platform. Once the code has been automatically generated from the models, i.e., the plant and the control models, it is deployed and executed on the corresponding hardware system, i.e., the real ECU and dSPACE SCALEXIO. In this study, a real-time system, i.e., the dSPACE MicroAutoBox II, is used as a real ECU and is connected to the dSPACE SCALEXIO via the CAN bus carrying the corresponding sensor and actuator signals, as is illustrated in Figure 7.

Thanks to having two control models, the softECU and the normal ECU model, the HIL system can be executed in two modes, namely the offline and online modes. Moreover, with the aim of covering the comprehensive characteristics of the engine system, several engine subsystems are modeled and simulated in detail, including the air path system, fuel system, piston engine system, exhaust system and cooler system. The implementation and execution of the experiments is supported by several software tools from dSPACE, namely ModelDesk, ConfigurationDesk and ControlDesk. Further information can be found in [100].

### 4.2. Experimental Setup

Despite the system complexity of the case study, thanks to the software tools provided by dSPACE, both configurations and parameterizations of the selected system with its components can be carried out in the user environment. Specifically, using ControlDesk, for example, the target sensors and communication signals can be selected, manipulated and acquired in real time. Thus, the user can configure the experiment, calibrate the controller, read off measurements and acquire data at the control station.

The configurations of the HIL experiment, in this study, are organized into three levels, namely the system configurations level, drive cycle configurations level and fault injector configurations level. At the system configurations level, the system parameters, including the internal and external system specifications, are specified. The key specifications of the present case study are presented in [21]. Setting the fault injector attributes, i.e., the fault type, fault time and fault location, enables the collection of a representative fault data set reflecting the system behavior in the presence of faults. Table 4 lists the essential specifications of the fault injector used to generate the data set as well as the number of the collected data samples in each class.

Based on the configurations of the fault injectors, different types of faults are injected at two locations, namely the accelerator pedal position (APP) sensor and the engine speed sensor. The sensors, into which the faults are injected, have been selected due to their significant impact on the vehicle behavior. According to a NASA investigation conducted in 2011, most of the accidents that have occurred in Toyota vehicles are caused by unintended acceleration [101]. On the other hand, the engine speed sensor has an important effect on all actuator control signals. Therefore, investigating the behavior of the system in the presence of faults on the selected sensors is vital.

**Figure 8:**
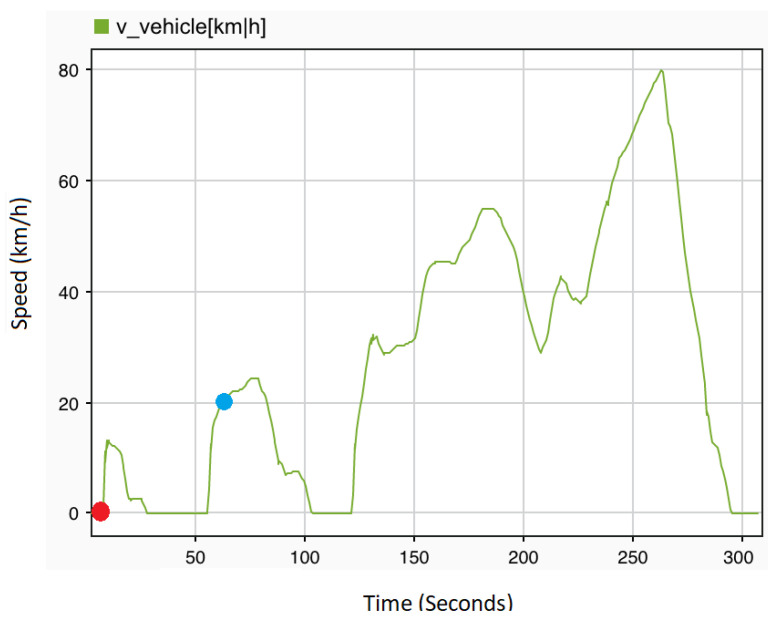
Driving cycle in dSPACE ControlDesk.

**Figure 9:**
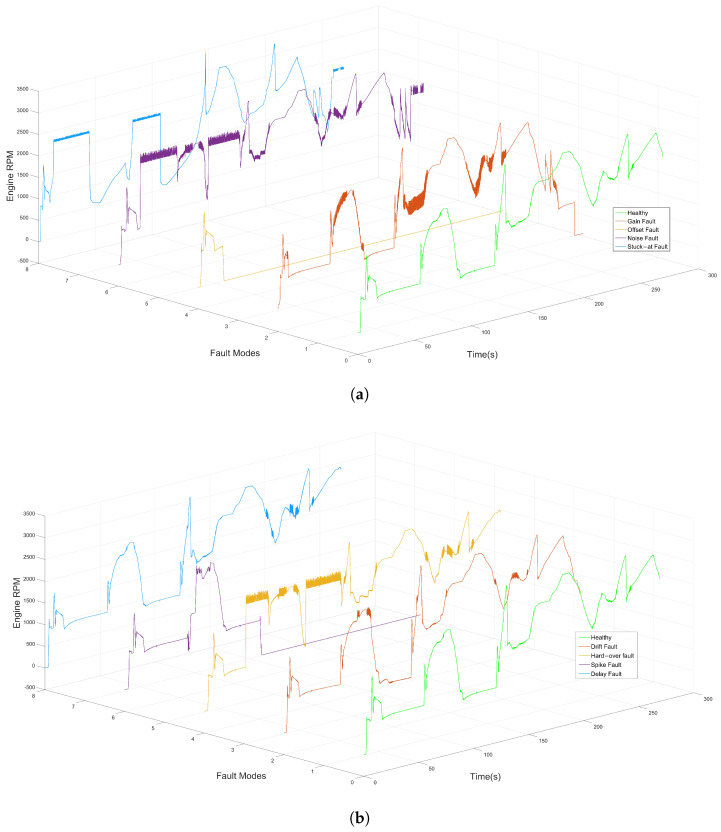
System behavior under fault conditions: (**a**) Gain, Offset, Noise, Stuck-at fault and Healthy signal. (**b**) Drift, Hard-over, Spike, Delay fault and Healthy signal.

For the setup of the driving scenario, ControlDesk is utilized to select and configure the driving cycle among a list provided by dSPACE, e.g., city and highway scenarios. On top of that, the execution of the selected driving scenario can be enabled in both automatic and manual modes in accordance with the user’s requirements. Figure 8 shows the selected driving scenario in this study. Note that the red dot indicates the starting point, and the blue dot indicates the current location. Figure 9 illustrates the system behavior in the presence of various fault types as a reaction to fault injection.

### 4.3. Data Set Description

During the experiments, the system behavior under normal and faulty conditions is recorded at the system level as time-series data, which serves as the basis for training and verifying the FDC model. The engine RPM, engine torque, vehicle speed, rail pressure and intake manifold pressure are the system-level variables considered for performing the detection and classification tasks. The recorded data are stored as an “IDV” file format. The data are then converted to a CSV format using the ControlDesk tool.

Once the records are captured, based on the proposed methodology, the data are pre-processed in order to clean, label, normalize and split them. However, the converted CSV file contains unusable data that could have a negative impact on the accuracy of the target model. Therefore, it is required to delete this kind of auxiliary information. During the labeling process, the fault types and the corresponding locations are assigned to the data. The labels are added to the aforementioned system variables in the form of numbers. Upon the completion of the labeling process, the HIL data are normalized using the Keras normalize function [102] to improve performance. By doing so, the data set is transformed into a binary matrix. Finally, the normalized data are split into three portions, one of which is used for training, the second for early model validation and tuning and the last for the testing and evaluation of the trained model. The distribution of the data is randomly determined, with 60% used for training and the remaining 40% is shared by both validation and testing, with 20% for each. After the cleaning steps, the total number of data points collected is 503,850. To be specific, 302,310 samples with 33,590 instances per class have been used for the training phase, while 101,670 samples have been used for the validation phase and the same number of samples have been employed as unseen data for the testing phase. Note that the unavailability of real-world automotive data sets to test the developed model is considered a threat to external validity. To address this threat in our experiments, unseen samples have been used as test data to verify the performance of the model.

### 4.4. Hyperparameters Optimization

**Figure 10:**
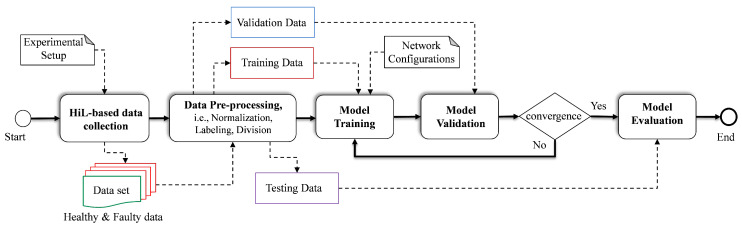
Flowchart of model implementation and optimization.

Following the data pre-processing, the next phase is the development of the FDC model, i.e., training and validation. Aiming at reducing the learning time of the training process, the implementation of the model is performed in the Google Colab environment, where Python is used with the Tensorflow [103] and Keras [104] libraries. Figure 10 shows the flowchart of the model implementation and optimization, initializing with the data collection and concluding with the testing of the optimally trained model.

To avoid the problem of overfitting, the trained model is validated using validation data, which in turn yields the model with the highest accuracy from the training data. In the course of the optimization process, the values of the hyperparameters are adjusted so that the optimal structure of the FDC model with the best performance can be obtained. In this regard, a set of hyperparameters, i.e., epochs, batch size, batch normalization layers, learning rate, CNN layers, LSTM layers, dense layers and drop layers, have been selected to be tuned according to the accuracy of each training. The range of the selected hyperparameters to be optimized is summarized in Table 5. Achieving the above goal requires the use of two evaluation metrics, namely accuracy and loss.

**Figure 11:**
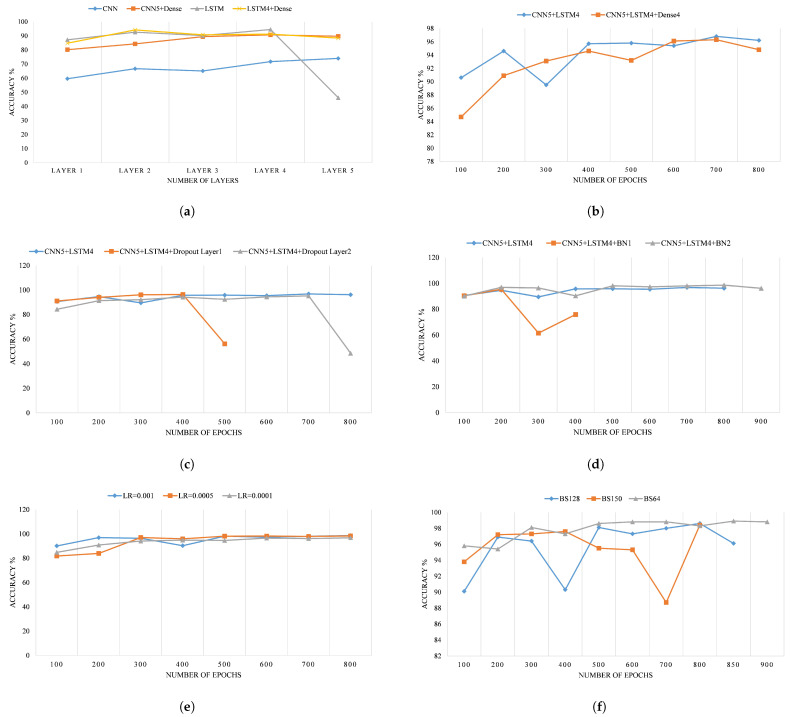
Hyperparameters optimization results. (**a**) Validation accuracy with different number of layers. (**b**) Validation accuracy with different number of epochs. (**c**) Validation accuracy with different number of dropout layers. (**d**) Validation accuracy with different number of batch normalization layers. (**e**) Validation accuracy with different number of learning rate. (**f**) Validation accuracy with different number of batch size.

These metrics are implemented in Python using the scikit-learn library [105]. The results of the optimization process in terms of accuracy are presented in Figure 11.

Based on the results presented in Figure 11a, the best validation accuracy of 90% was achieved by a five-layers CNN, including one dense layer. In contrast, no improvement in the validation accuracy was observed by adding more dense layers to the LSTM. Consequently, a five-layers CNN, a four-layers LSTM and four-layers dense are considered in the structure of the proposed model. Similarly, conducting the model training with different epochs shows that the best results, i.e., 96.8%, can be achieved with 700 epochs, as shown in Figure 11b. Another noteworthy point is that the performance of the model without dropouts is better than adding additional layers, as seen in Figure 11c. Therefore, the dropout layers are omitted in further experiments. On the other hand, due to the favorable validation results of the model, as a consequence of the addition of the batch normalization layers with a validation accuracy of 98.6% (Figure 11d), two batch normalization layers are added to the proposed structure. In terms of the learning rate, Figure 11e gives us an insight into the validation accuracy being improved by decreasing the learning rate; the best accuracy was achieved at 0.0005 with 98.3%. Finally, after determining the core hyperparameters, the model is trained with a varying number of batch sizes. In Figure 11f, it is noticeable that the results with batch size 64 are better than the others in terms of the validation criteria. As a conclusion of the validation phase, the optimized hyperparameters of the proposed model are presented in Table 6.

## 5. Results and Discussion

This chapter details the evaluation results of the optimized FDC model based on the test data along with a discussion of the capabilities of the proposed methodology to detect and classify different sensor and communication fault types. As stated in the methodology section, the model is evaluated based on various evaluation metrics, i.e., the accuracy, precision, recall and F1-score [99]. Moreover, the results of the proposed model are compared with standalone ML and DL techniques. In particular, the performance of the proposed model has been compared with two different single classifiers, namely LSTM and CNN.

### 5.1. Evaluation Metrics

Among the various available evaluation metrics, the focus of this study is on the accuracy, precision, recall and F1-scores. The classification accuracy, as one of the core metrics, is the ratio of the number of the correct predictions to the total number of input samples, as shown in Equation (9).

(9) $$Accuracy=\frac{TP+TN}{TP+TN+FP+FN}$$

where TP signifies the number of positive cases, i.e., the predicted fault types being correctly classified; TN describes the truly classified negative cases or the element dismissed correctly during classification; FP describes the number of cases misclassified as positive, also known as false alarm; and FN denotes the number of positive cases erroneously classified as negative or the unclassified faults.

The precision, or positive predictive value (PPV), on the other hand, indicates the proportion of the total classification results that are predicted to be correct. Mathematically, it can be calculated by dividing the number of true positive predictions by the sum of true positive and false positive predictions, as illustrated in Equation (10).

(10) $$Precision=\frac{TP}{TP+FP}$$

The recall, or true positive rate (TPR), is another metric that represents the sensitivity, indicating the percentage of correctly identified items compared to all items requiring identification. According to Equation (11), it can be calculated by dividing the number of true positives by the sum of true positives and false negatives.

(11) $$Recall=\frac{TP}{TP+FN}$$

Finally, the F1-score is the harmonic mean between precision and recall. As such, it allows the evaluation of the model in terms of precision and robustness. Mathematically, it can be calculated with Equation (12).

(12) $$F1\text{-}\mathrm{Score}=2*\frac{1}{\frac{1}{Precision}+\frac{1}{Recall}}$$

### 5.2. Testing Results

Based on the optimization results obtained in the previous phase, the hyperparameters have been tuned so that the optimal FDC model can be obtained. As a comparison with the individual LSTM and CNN models, Table 7 represents the detection accuracies of the proposed model according to the evaluation metrics. The results shows that the proposed model outperforms the standalone models in terms of the precision, recall and F1-score.

**Figure 12:**
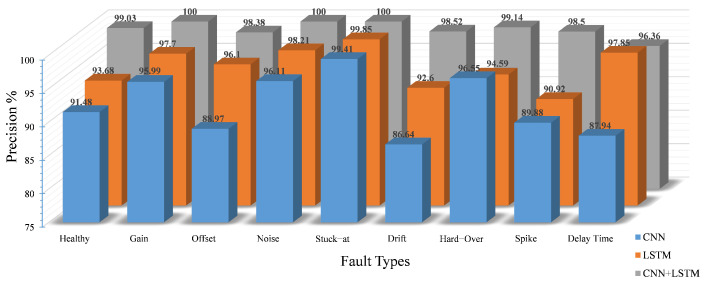
Classification performance in terms of precision.

**Figure 13:**
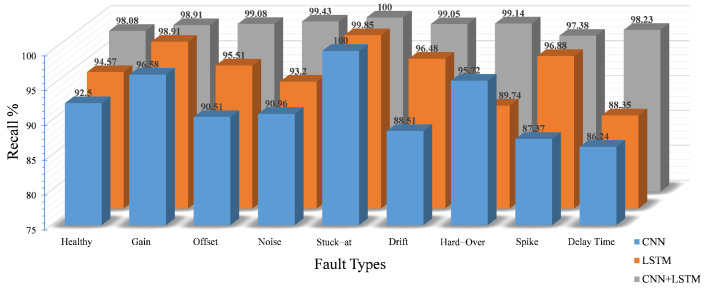
Classification performance in terms of recall.

**Figure 14:**
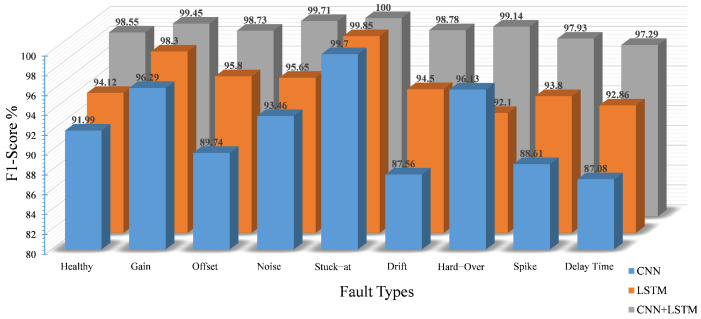
Classification performance in terms of F1-score.

For the classification of the detected faults, the performance of the proposed model over eight classes of faults plus healthy data in terms of precision, recall and F1-score is presented in Figure 12, Figure 13 and Figure 14, respectively. Generally, all networks have reasonable classification accuracy with unseen testing data, i.e., CNN with an average accuracy of 91.7%, LSTM with 95.6% and CNN-LSTM with 98.8%. Although the optimization process has been applied on the three DL models, it is noticeable that the proposed model as a hybrid architecture outperforms the standalone models.

In particular, concerning the precision score, the CNN model achieved a lower accuracy compared to the LSTM and the hybrid CNN-LSTM, especially for the drift, delay and offset faults, with a precision score of 86.64%, 87.94% and 88.98%, respectively. Because the number of false positives and false negatives increases with the healthy class in the CNN model, the probability of the false alarm rate also increases. On the other hand, the LSTM results exhibit an improvement in the performance, where the LSTM model outperforms the CNN-based model in terms of precision. The best precision score of the model is reflected in the stuck-at, noise, gain and delay faults, with accuracies of 99.85%, 98.21%, 97.7% and 97.85%, respectively. However, the limitations on the remaining fault classes have been overcome by our proposed model with an average accuracy of 98.8%. In addition, it is noticeable that the precision score is 100% for three classes, namely gain, noise and stuck-at, and is above 98% for the remaining classes, except for the delay class, which reaches 96.36%. The reason behind this limitation is that some features of the delay fault samples are similar to other classes and are incorrectly predicted as delay fault samples.

Observing the recall score of the aforementioned models, the individual CNN performance is still not satisfactory with an average accuracy of 95.6%, which is caused by the low recall score of the delay, spike and drift faults with 86.24%, 87.37% and 88.51%, respectively. By contrast, in the LSTM model, the recall value of seven classes is also above 90%. Compared to the CNN, the false positives for the healthy classes are relatively lower. Nevertheless, the percentage of false positives is 6.32%, and the percentage of false negatives is 5.92%. The probability of false positives for healthy data is still quite high. Therefore, in terms of the precision and recall of the LSTM model, the results are well suited for fault classification but not for fault detection due to the higher number of false positives and false negatives in the healthy class. However, the recall results of our proposed model are promising with good results for all target classes; the recall score is more than 98% for eight out of nine classes, except for spike faults with 97.38%.

As a harmonic mean, the combination of recall and precision is represented by the F1-score. The superiority of our proposed model is reflected in the values of the F1-score for each class compared to the other models (see Figure 14). The proposed model achieved very good accuracy with a minimum F1-score of 97.21%, outperforming the other models, i.e., the CNN and LSTM with minimum F1-scores of 87.08% and 92.1%, respectively.

**Figure 15:**
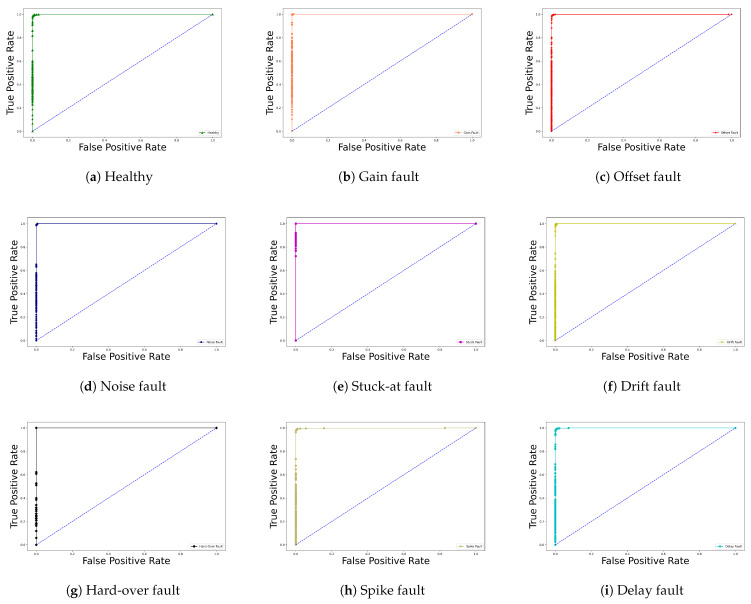
Classification performance in terms of AUC–ROC curve.

To illustrate the relationship between the true positive rate (TPR) and the false positive rate (FPR) of the proposed model, the classification results in terms of Receiver Operating Characteristic (ROC) curves are shown in Figure 15. The higher the ROC curve reaches the left corner, the better the classification performance. Accordingly, the results show that the proposed model exhibits a low false positive rate.

In summary, the hybrid CNN-LSTM-based FDC model with our proposed methodology surpasses other standalone DL techniques. With an accuracy of 98.5% and an F-score of 98.84%, respectively, in our proposed model, which are higher than other classification models with different classes of faults, the hybrid technique significantly excels most common state-of-the-art models. Subsequently, on the basis of proper integration, this can pave the way in the future for the development of more accurate FDC models.

### 5.3. Computational Complexity Analysis

In any application of an intelligent FDD model, besides the importance of prediction accuracy, the cost of training and inference should also be considered in terms of the time required. In this study, both training and inference times (in seconds) have been investigated, as shown in Table 8. As mentioned in the training section, cloud-based tools with high resources performance, i.e., Google Colab, were used as the development environment for the model.

Despite the high classification accuracy, the hybrid method requires a high training time. The reason is that the complexity of the model architecture is higher compared to the single model. On the other hand, the required execution time of the proposed model for classification under test data is significantly shorter than the LSTM model.

Compared to traditional ML methods, which require additional time for feature extraction, the time consumption of the proposed model is acceptable. Moreover, the target model can be trained offline once, but the real-time conditions for executing the model on a real-time system should be ensured. To accelerate the inference time, utilizing dedicated software libraries or selecting a specific network architecture such as PCA-GRU would be a good alternative. Based on the comparison, the trade-off between ensuring accuracy and reducing the complexity of the trained model paves the way for further improvements in the future to reduce computation time in real-time applications.

## 6. Conclusions

In this article, a novel effective and intelligent methodology for the fault detection and classification of an ASS during the development process, i.e., the system integration phase, is proposed. It aims at detecting and identifying system-level fault types, as a classification problem, in sensors and communication signals during the HIL testing process. For the purpose of designing a robust and accurate classification model, a hybrid CNN-LSTM network structure has been chosen among the DL techniques. By doing so, the limitations of each method can be overcome, and the advantages can be leveraged. To address the problem of a lack of representative training data, a novel real-time FI framework is employed with the HIL simulation platform through which eight different types of faults have been injected into the target components, i.e., sensor signals, along with complex test scenarios in real time. Consequently, the effects of the faults on the system behavior, in the form of time-series data, have been collected and used for the training, validation and testing of the target classifier. From a novelty standpoint, eight different types of sensors and communication faults have been programmatically injected in real-time without changing the original system model. In addition, high-fidelity simulations of the vehicle system involving realistic driving scenarios have been conducted, encompassing the entire vehicle model, i.e., the vehicle dynamics, environment, powertrain and engine model. Thus, the behavioral characteristics of the system can be accurately and comprehensively represented. Detailed phases of the proposed methodology for developing the FDC model have been presented, highlighting the key steps of the data set acquisition, data set pre-processing, model training, model validation and model testing.

For demonstrating the applicability of the proposed methodology and evaluating the performance of the proposed FDC model, a complex gasoline engine system has been used along with whole-vehicle and environment models. In this study, 60% of the collected data set is used for the training phase, while the remaining 40% is used for validation and testing, respectively. To improve the convergence rate of the proposed structure and to avoid the problem of overfitting, the optimization process is carried out in a manner that allows for an effective tuning of the hyperparameters. To analyze the performance of the proposed FDC model, different evaluation metrics have been used, i.e., recall, precision, F1-score and accuracy. Compared to standalone DL techniques, the experimental results demonstrate the superiority of our proposed model in terms of the classification accuracy on unseen test data, i.e., with an average accuracy of 98.85%. Specifically, the highest precision and recall scores have been obtained with our proposed model for eight different classes of faults along with the healthy class, with an average value of 98.88% and 98.81%, respectively. Additionally, the proposed model achieved a maximum F1-Score of 98.84%, outperforming the individual DL methods. Thus, from the results of our study, it can be concluded that the proposed model as a hybrid architecture surpassed the standalone models. Finally, the training and inference times of the models have been analyzed with the aim of highlighting avenues for further improvement.

Toward future lines of research, the findings imply that our proposed methodology can also be useful for analyzing recordings of real test drives conducted on public roads, so that unexpected faults can be intelligently detected and classified without human intervention. To this end, during the development of the FDC model, uncertain and imbalanced data, including unknown fault types, should be considered as potential further research in advanced feature engineering approaches. In the same way, as a future scope, considerable attention should be paid to the noise contained in real test drive recordings, which requires further development of the proposed model to improve the performance in the presence of high noise. On the other hand, developing a fault isolation model capable of determining the faulty components in complex systems with heterogeneous elements needs to be further investigated. Finally, to achieve full coverage of the faulty representative data set, future research will be conducted considering concurrent transient faults.

## Figures and Tables

**Table 1:** Value of $d_{v}$ and $o_{v}$ for all fault types.

Fault Type	$d_{v}$ Value	$o_{v}$ Value
Healthy Signal	1	0
Stuck-at Fault	0	0 or 1, and it varies with time
Offset/Bias Fault	1	fixed constant value
Gain Fault	Greater than 1	0
Noise Fault	1	random value
Hard-Over Fault	0	higher than maximum threshold
Spike Fault	1	value varies on time
Drift Fault	1	value increases on time
Delay Time Fault	0	last cycle value of x(t) based on time given

**Table 2:** Overview of the related work.

Related Works	Application Domain	Tasks	Method	Collected Data Set	Fault Classes	Remarks
Safavi et al. [77]	Autonomous vehicles	Fault detection, isolation, identification and prediction	Multiclass deep neural network (DNN)	Real autonomous driving data set	Four	High accuracy 99.84% Low fault coverage High data acquisition cost
Theissler [78]	Analyze automotive test recordings	Anomaly detection	Ensemble classifier-based ML	Real driving data set	Two	Low accuracy 85% Low fault coverage High data acquisition cost
Jung [79]	Combustion engine	Fault classification	Bayesian-based method	Real engine data set	Eight	Low accuracy 85% High fault coverage High data acquisition cost
Kaplan et al. [80]	Electric vehicles (EVs)	Fault diagnosis	LSTM	Simulation and real data set	Four	High Accuracy 97% Low fault coverage Low data acquisition cost
Zhong et al. [81]	Automotive engines	Simultaneous fault diagnosis	Probabilistic reject machine	Real data set	Ten	Low accuracy 88.74% Low fault coverage Low data acquisition cost
Biddle et al. [82]	Autonomous vehicles	Fault detection, identification and prediction	SVM	Simulation data set	Five	High accuracy 97.01% Low fault coverage Low data acquisition cost
Garramiola et al. [84]	Railway traction drives	Fault detection and isolation	Observer-based, frequency analysis and hardware redundancy	Real-time simulation data set	Two	Low accuracy Low fault coverage Low data acquisition cost
Raveendran et al. [85]	Air brake system	Fault identification	Random forest	Real-time simulation data set	Two	Low accuracy 92% Low fault coverage Low data acquisition cost
Namburu et al. [86]	Automotive engines	Fault diagnosis	Pattern recognition	Real-time simulation data set	Eight	High accuracy 99.25% High fault coverage Low data acquisition cost
Proposed Model	Automotive software systems development	Fault detection and classification	Hybrid CNN-LSTM	Real-time simulation data set	Eight	High accuracy 98.85% High fault coverage Low data acquisition cost

**Table 3:** The architecture parameters of the proposed model.

Layer	Size and Parameters
CNN Layer 1	Filters: 8, Kernel Size: 2, Activation: ReLU, Input Shape: [30 × 5], Output Shape: [30 × 8]
CNN Layer 2	Filters: 8, Kernel Size: 2, Activation: ReLU, Output Shape: [30 × 8]
CNN Layer 3	Filters: 8, Kernel Size: 2, Activation: ReLU, Output Shape: [30 × 8]
CNN Layer 4	Filters: 8, Kernel Size: 2, Activation: ReLU, Output Shape: [30 × 8]
CNN Layer 5	Filters: 8, Kernel Size: 2, Activation: ReLU, Output Shape: [30 × 8]
Batch Normalization Layer 1	Output Shape: [30 × 8]
Max Pooling Layer	Pool Size: 2, Output Shape: [15 × 8]
LSTM Layer 1	Neurons: 64, Activation: ReLU, Output Shape: [15 × 64]
LSTM Layer 2	Neurons: 64, Activation: ReLU, Output Shape: [15 × 64]
LSTM Layer 3	Neurons: 64, Activation: ReLU, Output Shape: [15 × 64]
LSTM Layer 4	Neurons: 64, Activation: ReLU, Output Shape: 64
Batch Normalization Layer 2	Output Shape: 64
Flatten Layer	Output Shape: 64
Output Layer	Output Shape: 9

**Table 4:** Configuration of fault injection with collected data samples.

Fault Type	APP Value	RPM Value	Fault Duration	Data Samples
Gain	10	5	0–300	59,945
Offset	5	300	0–300	63,910
Noise	1–100	0–8191	123–288	46,115
Stuck-at	0	0	0–300	59,830
Drift	0.1	10	17–86, 44–100, 122–300	60,195
Hard-Over	127	10,000	122–135, 184–225	34,800
Spike	1–100	700–8191	0–96, 103–293	57,805
Delay	10	3	0–96, 121–286	55,905

**Table 5:** Range of hyperparameters used in optimization process.

Hyperparameter	Range
CNN Layers	0–5
LSTM Layers	0–5
Dense Layers	0–5
Epochs	50–900
Max Pooling Layer	0–1
Drop Layer	0–2
Batch Normalization Layer	0–2
Batch Size	64–150
Learning Rate	0.001–0.0001

**Table 6:** Optimal Hyperparameters for CNN+LSTM Model.

Hyperparameter	Value
CNN Layers	5
LSTM Layers	4
Dense Layers	0
Epochs	850
Max Pooling Layer	1
Drop Layer	0
Batch Normalization Layer	2
Batch Size	64
Learning Rate	0.0005

**Table 7:** Fault detection accuracies of the models.

Model	Precision	Recall	F1-Score
CNN	92.69%	91.99%	92.32%
LSTM	95.98%	94.87%	95.36%
CNN-LSTM	98.86%	98.90%	98.88%

**Table 8:** Training and inference times of the models.

Model	Training Time (s)	Inference Time (s)
CNN	368.4	1.14
LSTM	4874.7	5.22
CNN-LSTM	9541.2	3.07

## Data Availability

Data available on request due to restrictions.
